# Application of a self-injection locked cyan laser for Barium ion cooling and spectroscopy

**DOI:** 10.1038/s41598-020-73373-w

**Published:** 2020-10-05

**Authors:** Anatoliy A. Savchenkov, Justin E. Christensen, David Hucul, Wesley C. Campbell, Eric R. Hudson, Skip Williams, Andrey B. Matsko

**Affiliations:** 1 OEwaves Inc., 465 North Halstead Street, Suite 140, Pasadena, CA 91107 USA; 2 Department of Physics and Astronomy, University of California-Los Angeles, Los Angeles, CA 90095 USA; 3 United States Air Force Research Laboratory, Rome, NY 13441 USA; 4 Innovare Advancement Center, Rome, NY 13441 USA; 5 UCLA Center for Quantum Science and Engineering, Los Angeles, CA 90095 USA; 6 Jet Propulsion Laboratory, California Institute of Technology, 4800 Oak Grove Drive, Pasadena, CA 91109-8099 USA

**Keywords:** Lasers, LEDs and light sources, Atomic and molecular interactions with photons, Ultracold gases

## Abstract

Compact, high power lasers with narrow linewidth are important tools for the manipulation of quantum systems. We demonstrate a compact, self-injection locked, Fabry-Perot semiconductor laser diode with high output power at 493 nm. A high quality factor magnesium fluoride whispering gallery mode resonator enables both high passive stability and 1 kHz instantaneous linewidth. We use this laser for laser-cooling, in-situ isotope purifcation, and probing barium atomic ions confined in a radio-frequency ion trap. The results here demonstrate the suitability of these lasers in trapped ion quantum information processing and for probing weak coherent optical transitions.

## Introduction

Lasers with high optical power and narrow linewidth are useful probes for the manipulation of coherent, quantum systems. As the output power increases and the linewidth narrows, quantum systems composed of increasing numbers of particles can be coherently probed more accurately for a longer duration of time. Whispering gallery mode (WGM) lasers with small, high quality factor cavities have been used to make self-injection locked lasers in the infrared spectrum with integral linewidths of order of 100 Hz^1^. However, many candidate qubit systems, such as trapped atomic ions and nitrogen vacancy centers, possess optical transitions in the visible and ultraviolet spectrum. Extending these narrow linewidth WGM lasers from the infrared spectrum into the visible and ultraviolet optical spectrum at high optical power has proved challenging due to nonlinear absorption effects in the laser^2,3^. Here we extend the design of WGM lasers to operate at high power ($\sim$ 100 mW) with narrow instantaneous linewidth (1 kHz) while addressing a visible optical transition in a trapped barium ion near 493 nm. The simultaneous high power and low linewidth visible wavelength laser was achieved by rarefying the available optical modes of the WGM cavity and controlling the amount of optical feedback to the laser diode. The type of laser presented here should have wide applicability for laser cooling, addressing narrow optical transitions, probing optical clock transitions in atoms, and controlling atomic and some solid state qubit systems^1,2,4–11^. In addition, this WGM laser can be packaged in a small form factor and thus could have lower sensitivity to environmental noise than standard external cavity diode laser designs.

A narrow linewidth laser can be constructed using a laser diode coupled to a high-quality factor (Q-factor) resonator. Optical feedback to the laser diode is provided by the small, coherent backscattering from the WGM optical modes. The feedback collapses the spectrum of the laser diode to a high Q-factor optical mode. This self-injection locking mechanism^4^ has produced narrow linewidth lasers from the blue part of the visible spectrum at low power^2^ to the mid-infrared part of the spectrum at high power^3^. Operating these lasers in the visible or ultraviolet at high power and low linewidth is challenging due to non-linear optical effects such as stimulated Raman and Brillouin scattering^12,13^ and hyperparametric instability^14^. The optical power threshold for the significant onset of these deleterious effects is proportional to $1/Q^2$, competing with the $1/Q^2$ scaling of the laser linewidth. As a result, the Q-factor of the resonator should be chosen to balance linewidth and optical power requirements.

**Figure 1:**
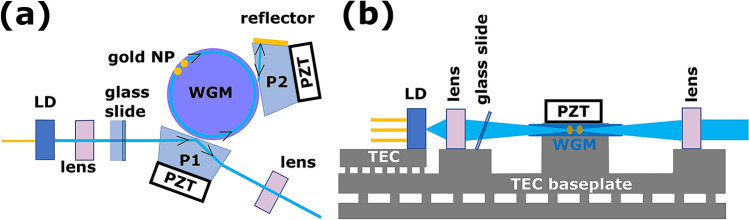
Diagrams of self-injection laser to illustrate principle of operation. (**a**) Top view. Light from a single mode Fabry Perot laser diode (LD) is focused with a lens and sent through an adjustable glass slide into an anti-reflection coated coupling prism (P1) made out of BK7. The glass slide, mounted on a tilt stage, compensates for mechanical creep. A piezo-electric transducer (PZT) adjusts the distance between P1 and the whispering gallery mode laser (WGM) cavity to control the evanescent coupling into laser cavity and to ensure single-mode operation^20^. Light circulates in the WGM cavity and the resonant modes are controlled by the geometry of the cavity. The reflective gold nano-particle (NP) size and spacing aid in rarefying the resonant modes in the cavity. A PZT on top of the WGM cavity, not shown in the illustration, controls the wavelength of the light by applying a force on the WGM cavity to change its refractive index [see (**b**)]. A third PZT controls the distance between the WGM and the reflector prism (P2) to adjust the amount of optical feedback into the laser diode. Light from the WGM evanescently couples into P2, and a gold-coated face of P2 reflects light back into the laser diode. The laser output exits P1 where it is collimated by a lens. (**b**) Side view with P1, P2, and associated PZTs not shown. The temperature of the Fabry Perot laser diode and optical bench are controlled by thermo-electric cooler (TEC) baseplates. The LD focusing lens is mounted on a five axis stage (not shown in the figure for clarity) for beam steering and adjusting mode-matching into the WGM cavity.

The density of the optical spectrum impacts the self-injection locking dynamic range. A WGM cavity lasing at wavelength $\lambda$ has optical spectrum density increasing faster than $1/\lambda ^2$, so WGM modes at visible and ultraviolet wavelengths are more susceptible to mode competition which disrupts the locking dynamic range. The optical spectrum density can be rarefied by reducing the WGM size, but reduced mode volume reduces thresholds for nonlinear processes like stimulated Raman scattering and four wave mixing. Instead, we used a larger WGM cavity (diameter 1.83 mm) to avoid these nonlinear processes while simultaneously adding gold nano-particles to the cavity to rarefy the optical spectrum density^15^. This technique enables high power single mode operation while maintaining the dynamic locking range of the laser (see Fig. 1).

In what follows we describe the self-injection locked 493 nm WGM laser architecture, present the results of laser characterization, and demonstrate usability of the laser for cooling and trapping atomic Ba$^+$ ions. Trapped barium ions are attractive qubits because they require only visible lasers for laser cooling, state preparation and measurement, and manipulation of the qubits in the $^2$S$_{1/2}$ ground state. Additionally, barium ions have long-lived metastable excited electronic states ($\tau \approx$ 30 s and 80 s^16^), making trapped barium ions candidate optical clock qubits^17,18^. A recent demonstration of a hyperfine qubit in the A = 133 radio-isotope of barium resulted in the lowest state preparation and measurement error of any qubit^19^. The successful demonstration of a compact, self-injection locked 493 nm laser paves the way towards laser modules suitable for deployable trapped ion quantum systems.

## Results

### Experimental setup

The principle of operation of the laser system shown here is similar to a standard external cavity diode laser (see Fig. 1)—a laser diode is coupled to a narrowband cavity, and optical feedback to the diode selects the lasing mode. The optical path length from laser diode to the WGM cavity and from the WGM to the reflector is short to reduce technical fluctuations of the phase of the feedback. The principle of operation of the laser is given in the caption of Fig. 1.

Temperature control of the laser provides passive stability to this small footprint laser, so the single spatial mode Fabry-Perot laser diode (Nichia) is enclosed in a sealed TO-9 package with an anti-reflection coated window. The diode package contains an internal tracking photodiode coupled to the laser diode via its back facet, and the laser diode mount has a thermistor and Peltier element for closed-loop thermal control. The 1.83 mm diameter MgF$_2$ WGM cavity is mounted on a custom, miniaturised, thermally controlled platform matched to the thermal expansion of the resonator. The platform is equipped with a thermistor and a miniature Peltier element for closed-loop temperature stabilization of the WGM cavity.

Because short-wavelength laser diodes have high divergence angles, mode-matching the laser output into a small-aperture WGM cavity requires an optical system with high magnification. The optical coupling of the laser emission to the WGM cavity is sensitive to wavelength-scale displacements of the optical components, and mechanical creep of the laser components results in a significant degradation of the coupling. Adjusting the yaw and pitch of a thin, glass slide inclined at Brewster’s angle compensates for the long term mechanical creep of thermal aging of the optical components (see Fig. 1). The 3.5 mm thick glass is thick enough to displace the beam without significant introduction of optical aberrations affecting coupling into the WGM cavity. A five-axis stage controls the position and angles of the focusing asphere lens and compensates for coma and astigmatism of the laser diode package’s view port.

The laser here produced approximately 100 mW of narrow linewidth laser light near 493 nm when coupled to the WGM cavity. When not coupled to the WGM cavity, the same laser diode produced approximately 120 mW of light.

### Self-injection locking of a high power laser

**Figure 2:**
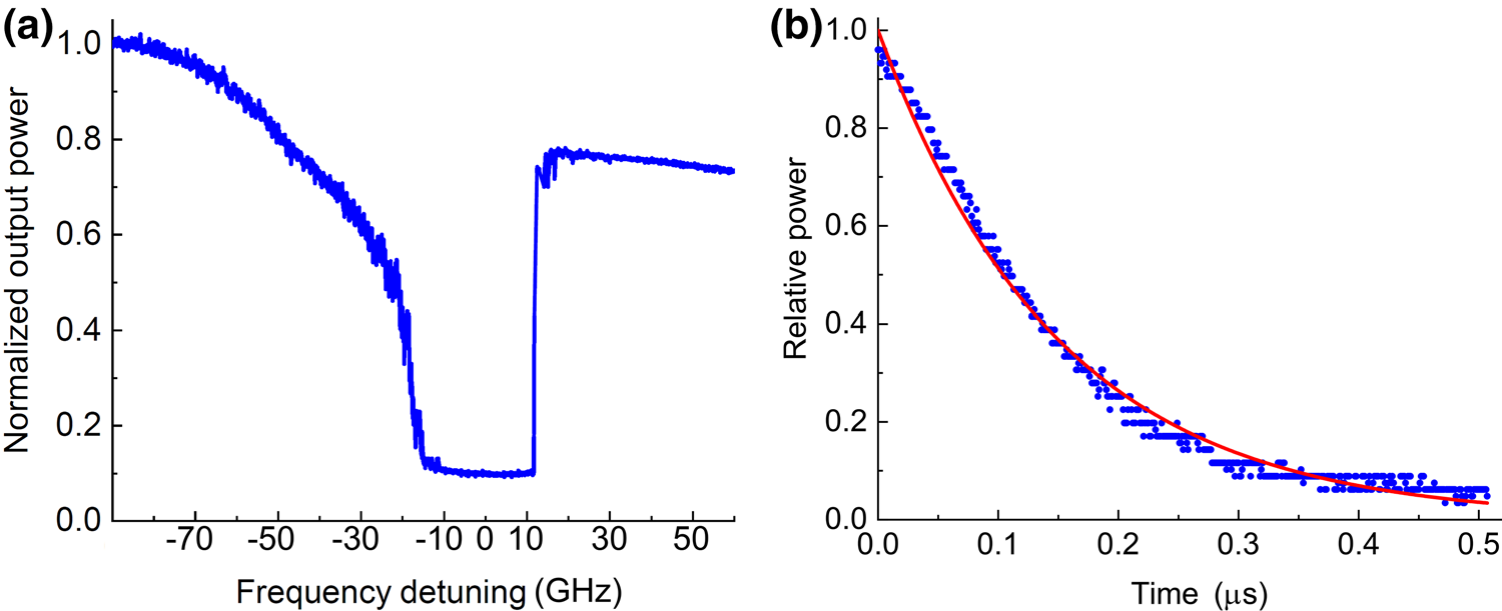
Output power and cavity ringdown of a high power WGM laser near 493 nm. (**a**) Dependence of the output laser power on the detuning from resonance at low laser power. The laser current controls detuning from the laser cavity resonance. The laser light near $$f_\text {laser} = 607$$ THz does not enter the WGM cavity if the fabry perot mode is detuned from the WGM with bandwidth $$f_\text {laser} / Q_{int} =$$ 607 THz/10$$^9$$$$\approx $$ 607 kHz. The optical power then exits the laser assembly (see Fig. 1) with large linewidth. As the detuning becomes smaller at low optical power, up to 90% of the non-resonant light enters WGM cavity and is absorbed or reflected back to the laser, reducing the output power of the laser. Self-injection locking is sustained here, and the figure shows a dynamic locking range of approximately 20 GHz. The output laser power has linewidth much less than the bandwidth of the WGM cavity. (**b**) Ring-down signal from the WGM cavity while coupling to a higher order mode at high power. The ring-down time of 0.17 $$\upmu $$s corresponds to a loaded Q-factor of $$2 \times 10^6$$. The internal Q-factor $$Q_{int}$$ of the resonator, inferred from the measured contrast from an optical spectrum analyzer with low-power laser operation, exceeds $$10^9$$.

**Figure 3:**
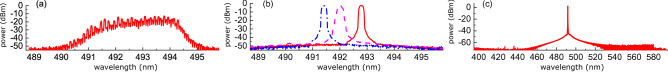
Spectrum of a free running laser diode and spectrum of laser diode coupled to a WGM cavity. (**a**) Spectrum of a single mode free-running Fabry-Perot laser used in the experiment. (**b**) Spectra of the self-injection locked Fabry-Perot laser taken using an optical spectrum analyzer (OSA). The laser is locked to several different WGM modes spanning most of the emission bandwidth of the laser diode. The linewidth is limited by the resolution of the OSA. (**c**) Optical spectrum taken in a broader spectral window for one of the modes used in (**b**). No significant spurious cavity harmonics are observed.

The free-running diode laser has a longitudinal mode spacing of approximately 84 GHz (Fig. 3a), and the laser operates in the self-injection locked regime when one of these modes is tuned within the vicinity of a WGM cavity mode, reflecting light back to the Fabry-Perot laser diode. Self-injection locking occurs when the relative reflection exceeds approximately 0.1% and is sustained when the laser frequency detuning from the cavity mode is less than the full-width, half maximum of the WGM cavity (see Fig. 2a). Like a standard external cavity diode laser, a self-injection locked WGM laser significantly reduces the linewidth of the laser diode, becoming much narrower than the linewidth of the cavity resonator mode^1,2,4–11^.

At high optical power, overcoupling the WGM cavity avoids thermal and optical instabilities while still producing narrow linewidth laser light. Standard cavity ringdown techniques can be used to measure the Q-factor of the laser^2^, however, in this laser geometry, the pump laser and light exiting the cavity share a common optical path. The laser Q-factor was instead measured using a higher order cavity mode to create a geometrical separation between the pump light and light exiting the cavity. We measure a Q-factor of $2\times 10^6$ (see Fig. 2b) at high output power.

When critically coupled, the WGM spectrum has four mode families with modes spaced by $\simeq 10$ GHz with a contrast of at least 30%. To run the system at high power we decrease the air gap between the resonator and the coupling prism to over-couple the resonator modes. The contrast of the modes is reduced to a few percent- enough to support self-injection locking in principle. However, many of these over-coupled WGM modes do not reflect into the laser diode efficiently and the phase of the light returning to the laser from these modes do not support lasing^2,10,11^. Figure 3b shows three lasing modes each separated by $\sim 1$ THz, showing coverage of the free-running laser diode spectrum shown in Fig. 3a. Figure 3c illustrates the broadband spectral purity of the laser emission.

**Figure 4:**
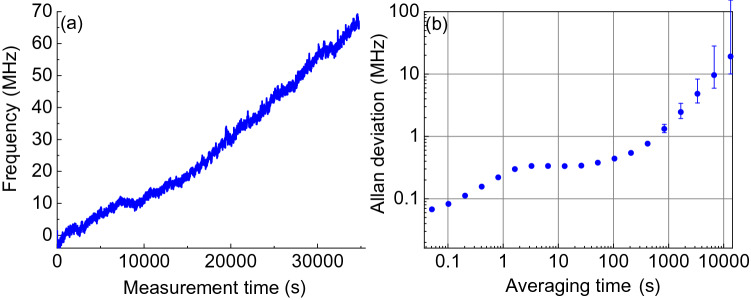
Passive stability of the laser emission near 608 THz. (**a**) Laser frequency near 608 THz with the initial frequency set to zero. The measured laser drift over $\approx 10^4$ s is of order the systematic frequency uncertainty of the wavemeter. (**b**) Allan deviation calculated for the data set (**a**) treating the wavemeter as a stable reference. The self-injection locked WGM laser is stable over short and long timescales.

The passive stability of this laser operated near 607 THz is shown in Fig. 4 without stabilization to a reference. The absolute laser frequency is queried every 100 ms with a High Finesse WSU-2 wavemeter with a systematic frequency uncertainty of ± 20 MHz. After $\approx 3\times 10^4$ s, the laser drifted $\approx 70$ MHz from its initial value. The Allan deviation of the laser in Fig. 4b shows the passive stability of the laser is of order the systematic uncertainty of the wavemeter over $\approx 10^4$ s. The laser passive stability can be significantly improved with better packaging of the device.

It is possible to estimate the linewidth of the laser by observing the drop in the output power of the laser assembly as light from the diode enters the WGM cavity (see Fig. 2a). As the laser enters the self-injection locked regime at low power, more than 90% of the optical power enters the WGM cavity. The laser linewidth $\Delta \nu$ can then be estimated from $\Delta \nu \approx \gamma \times (1-C)/2C$ where $C=(P_{in}-P_{out})/P_{in}$ for WGM cavity input and output powers $P_{in}$ and $P_{out}$ and $\gamma$ is the full-width half maximum bandwidth of the critically coupled WGM cavity^2^. Using $C=0.9$ and $\gamma = 600$ kHz, an estimate of the laser linewidth is $\Delta \nu \approx 30$ kHz.

**Figure 5:**
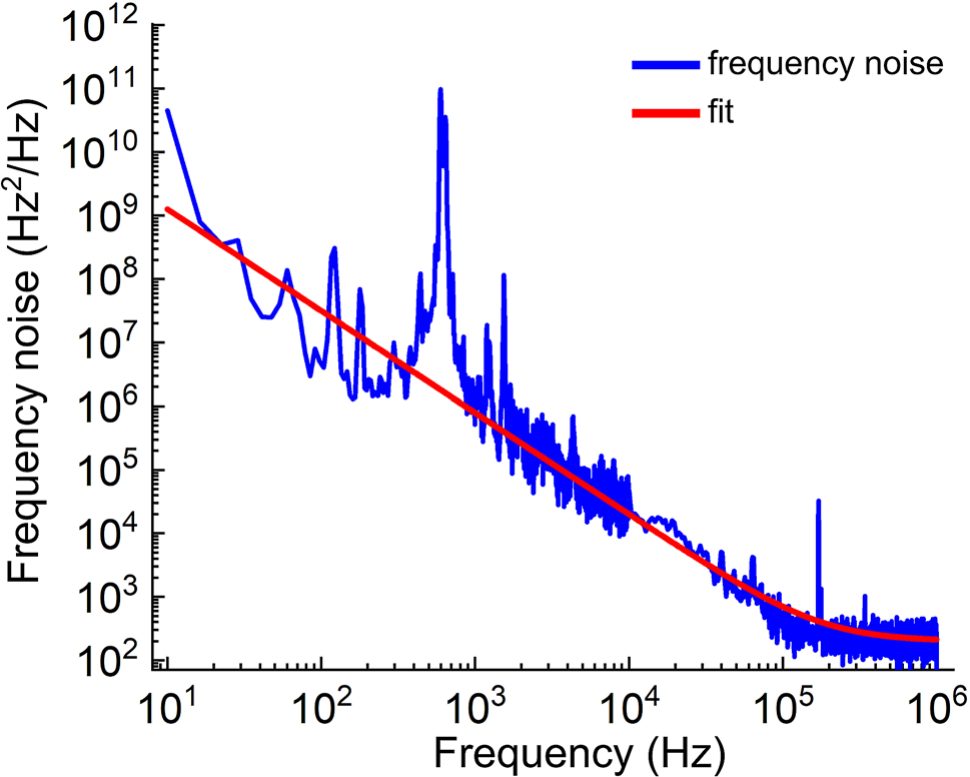
Frequency noise of the self-injection locked laser measured using Fabry-Perot etalon. The peaks near 800 Hz are measured mechanical modes of the etalon, and the laser frequency noise reaches the measurement noise floor of 300 Hz$^2$/Hz at approximately 100 kHz. The red line is a numerical fit to the data with a $f^{-1.6}$ frequency dependence. By integrating this fit, we find an effective linewidth of 14 kHz. The instantaneous linewidth limit of this laser, given by $\pi$ times the measurement noise floor^6^, is $\approx$ 1 kHz.

At high output power ($\approx 100$ mW), we measured the instantaneous linewidth of this laser using a Fabry-Perot etalon consisting of two 3 mm thick glass plates separated by 1 mm of air. When the laser frequency is tuned to the slope of the etalon transmission, the etalon transforms frequency noise to amplitude noise and is measured using a fast photodiode. The frequency noise shown in Fig. 5 can be used to plot the single side phase noise density^6^ which can be integrated to determine the effective laser linewidth. The effective linewidth shown here at 100 mW laser output near 493 nm is $\approx$ 14 kHz. The instantaneous linewidth is derived from the same plot by multiplying the noise floor in the figure by $\pi$, resulting instantaneous linewidth of 1 kHz^6^.

### Ion trapping experiment

**Figure 6:**
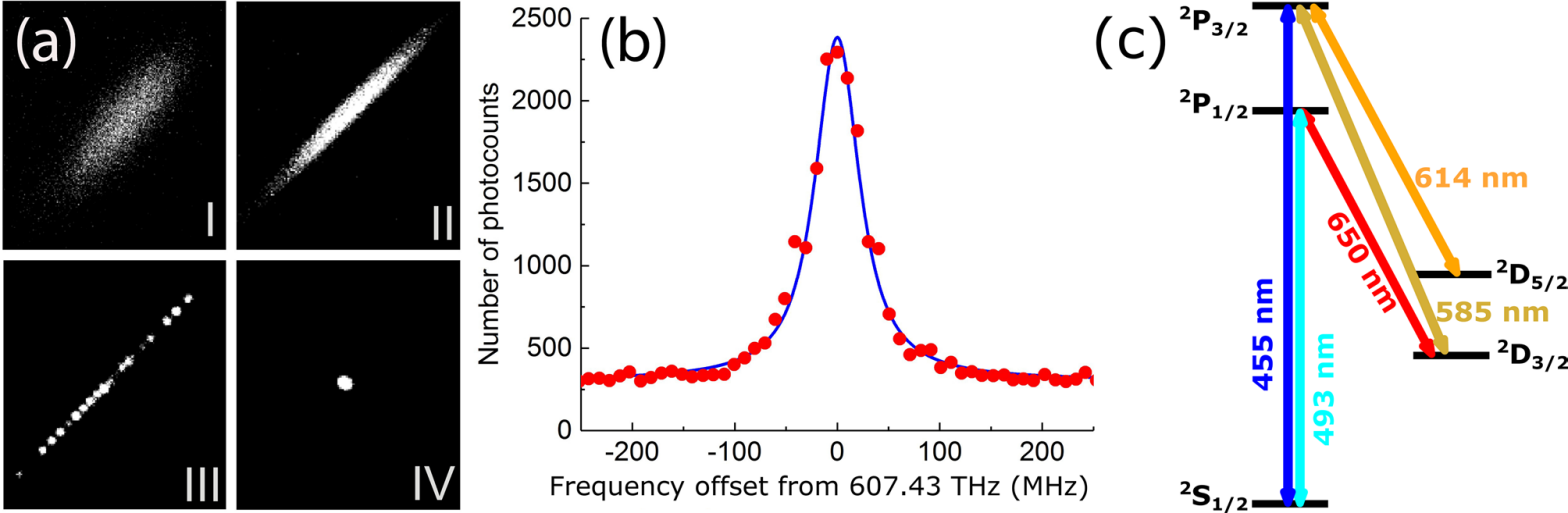
(**a**) Isotope purification and distillation of a single ${}^{134}\text {Ba}^+$ ion loaded via laser ablation from a natural abundance $\text {BaCl}_2$ source. Stage I is an image from an intensified CCD camera of the ion cloud after laser ablation^21^ and contains naturally occurring isotopes of barium atomic ions and BaCl molecular ions. Stage II depicts evolution of the ion cloud after filtering out BaCl molecular ions by applying a dc-quadrupole to the ion trap electrodes. Stage III is the resulting linear ion chain after lowering the amplitude of trap RF voltage. Stage IV is a single ${}^{134}\text {Ba}^+$ ion distilled from the longer chain in stage III. (**b**) Atomic spectrum of the $^{2}S_{1/2} \leftrightarrow ^{2}P_{1/2}$ transition in ${}^{134}\text {Ba}^+$ using a self-injection locked WGM laser near 493 nm. Using a fiber EOM, the frequency of the laser light near 493 nm (607 THz) is rapidly switched between laser cooling and probing of the atomic transition. Photons are counted using single photon counting photo multiplier tubes during the probe cycle resulting in the displayed spectrum. (**c**) Grotrian diagram of a barium atomic ion with nuclear spin $I = 0$ ($^{130,132,134,136,138}\text {Ba}^+$). The figure shows the electric dipole transitions used for laser cooling and electron shelving and de-shelving^19,21^. The narrow linewidth and high passive stability of the visible wavelength WGM laser could enable more robust operation of trapped ion qubits.

The barium ions used to test the laser are confined using a linear radio frequency (RF) Paul trap (Fig. 6a) operating with a peak-to-peak RF voltage of $V_{pp}= 200$ V at frequency $\Omega \approx 2\pi \times$1 MHz. The minimum distance between the trap axis and the electrodes is approximately 3 mm^19,21^. A self-injection locked laser near 493 nm described in this report and a grating-stabilized external cavity laser near 650 nm laser cools trapped atomic barium ions loaded by laser ablation of a BaCl$_2$ target. Light from both lasers pass through fiber electro-optic modulators (EOMs) with 6-GHz bandwidths to provide frequency sidebands on the lasers’ spectra, which allow for cooling and/or heating multiple isotopes simultaneously as well as for addressing the necessary transitions due to hyperfine structure^19,21^. This light is delivered to the ion trap vacuum chamber with circular polarization via single mode optical fibers and impinges on the trapped ions with a $45^{\circ }$ angle with respect to an applied magnetic field of a few Gauss used to destabilize dark states that result from coherent population trapping^22^.

Using the self-injection locked laser described above, the spectrum of the $^{2}$P$_{1/2} \leftrightarrow ^{2}$S$_{1/2}$ transition in $^{134}\mathrm {Ba}^+$ was measured near 493 nm, and is shown in Fig. 6. The frequency of the laser is monitored by a wavemeter with systematic uncertainty of $\sim$ 20 MHz, and the laser’s passive stability over timescales of $\sim$ 1 min allow for the acquisition of atomic spectra as depicted in Fig. 6b. The spectrum was obtained using a modular digital synthesis platform^23^ to rapidly alternate between Doppler cooling (100 $\upmu$s duration) and weak optical excitation (50 $\upmu$s duration) for fluorescence spectroscopy to prevent laser-induced lineshape distortions^21,24^. The transition near 493 nm has a natural linewidth of 20.1 MHz, and we observe broadening of this transition due to unresolved Zeeman structure and drift of the wavemeter used to stabilize the lasers. A fit to a Lorentzian lineshape gives a width of 57 MHz, comparable to the same measurements made using a standard Littrow laser^21^. Figure 6 shows the suitability of a WGM laser near 493 nm for laser-cooling, in-situ isotope purification, and spectroscopy of trapped atomic barium ions.

## Discussion

We have shown the operation of a high-power, visible wavelength laser with narrow linewidth and high passive stability suitable for laser cooling trapped ion qubits. The laser was produced using a single-mode Fabry-Perot laser diode self-injection locked to a WGM cavity. The large dynamic locking range of the multimode resonator should enable long term stabilization of the laser for probing highly-coherent quantum transitions.

Several factors in the laser design are necessary to maintain both high spectral purity and a large dynamic locking range. The shape of the resonator rarefies the optical spectrum to reduce the number of high order optical modes by damping them on the top and bottom surfaces of the WGM cavity. Reflective gold nano particles placed on the surfaces of the WGM cavity further induce feedback to the desired laser modes. Finally, the aspheric lenses efficiently couple the desired laser mode into the WGM cavity to further enhance optical feedback of the modes. In principle, it should be possible to create a single-mode resonator with only one mode family for each polarization to minimize linewidth and maximize the dynamic locking range.

The total volume of the laser assembly, excluding the optical isolator ($\sim 100$ cm$^3$), can approach $\sim 1$ cm$^3$, primarily limited by the need for thermal management of the TEC baseplates needed for temperature stabilization. A small, direct-diode laser package, like the one presented here, should have smaller vibration sensitivity and can potentially be useful on moving platforms.

## Methods

A high-power, single mode Fabry Perot laser diode in a standard TO-can package is attached to a stage and both the stage and diode mount are temperature stabilized to $\sim$100 mK (see Fig. 1). The laser diode can be swapped by replacing mounting screws without the need for wire bonding or soldering. An asphere objective with five-axis control directs focused light through an anti-reflection coated compensation window and into a coupling prism. A PZT controls the amount of light coupled into a whispering gallery mode of a MgF$_2$ toroidal microdisk resonator. This toroidal resonator does not have polar caps, and the edges are chamfered and coated with an optically absorbing material to provide additional control of the spectral density of modes. A piezo-actuator is attached at the top of the resonator to control the laser frequency. Approximately 100 mW of laser light near 493 nm exits the resonator, passes through and exits the coupling prism where the light can be focused into a fiber optic cable. This fiber optic cable is coupled to an EOM and then delivered to an ion trap through an acousto-optic modulator.

Optical feedback from the resonator is controlled in two ways. Two 100 nm gold nano-particles were deposited on the equator of the toroidal resonator approximately 250 $\upmu$m apart to induce amplified backscattering of the selected fundamental mode family while simultaneously decreasing the backscattering of higher order optical modes. The number of gold nano-particles in this work was limited by available fabrication capabilities, but proper placement of more gold nano-particles could provide more feedback of the fundamental optical mode. In addition, a second evanescently coupled prism (P2 in Fig. 1a) has a semi-transparent, gold-coated surface to provide additional feedback to the laser. A PZT controls the spacing between the WGM cavity and the prism P2 to adjust the amount of feedback to the laser.

The laser wavelength can be controlled in several ways. The asphere between the laser diode and toroidal resonator (see Fig. 1) is equipped with a heater that provides up to 10 ${^\circ }$C of temperature tuning to adjust the distance between the laser diode and the cavity. The laser diode TEC baseplate can heat or cool the laser diode to provide more control over the laser wavelength. Finally, the PZT on top of the WGM cavity provides high speed control of the laser wavelength.
